# Neuraxial Anesthesia in a Parturient With Newly Diagnosed Klippel-Trenaunay Syndrome: A Case Report

**DOI:** 10.7759/cureus.101521

**Published:** 2026-01-14

**Authors:** Michelle T Vo, Allen Wang, Andrew H Taniguchi

**Affiliations:** 1 Anesthesiology, University of California Davis Medical Center, Sacramento, USA

**Keywords:** cesarean section, high-risk obstetric anesthesia, klippel-trenaunay syndrome, neuraxial anesthesia, spine imaging

## Abstract

Klippel-Trenaunay syndrome (KTS) is a complex, congenital vascular disorder characterized by three primary manifestations: cutaneous capillary malformation (most commonly port-wine stain), congenital varicose veins, and soft tissue and bone hypertrophy. In the obstetric population, the evaluation of these vascular malformations is essential to mitigate bleeding risk with regional, neuraxial, and general anesthesia, particularly if there is the involvement of the spinal column or oropharyngeal airway. We report a unique case highlighting the successful administration of neuraxial anesthesia in a parturient with KTS undergoing an emergent cesarean section for fetal deterioration. In the absence of available imaging, careful consideration was given to balance the risks of neuraxial hematoma and a potentially difficult airway.

## Introduction

Klippel-Trenaunay syndrome (KTS) is a rare, vascular condition that is diagnosed based on the presence of two of the three physical examination findings: cutaneous vascular malformation, venous malformation, and the overgrowth of bone and soft tissues [1]. Most commonly, a cutaneous capillary malformation (typically presenting as a port-wine stain) is the earliest clinical manifestation of KTS present at birth, though significant heterogeneity exists among individuals with KTS regarding the presence and distribution of these capillary, venous, and, at times, lymphatic malformations [2]. The prevalence of KTS is estimated to be 2-5 in every 100,000 individuals [3]. For women with KTS, the condition can pose significant risks in pregnancy. This is largely attributed to the low-flow nature of these vascular malformations, which increases the risk of thromboembolic events in a pregnancy-induced hypercoagulable state, and the organ involvement of these malformations, which can increase the risk of hemorrhage. Horbach and colleagues observed the presence of deep vein thrombosis and pulmonary embolism in 5.8% and 2.3% of KTS pregnancies, respectively, presenting a 100-fold increase in relative risk compared to the general population (p < 0.0001) [4]. Postpartum hemorrhage risk is also elevated due to venous malformations that can be present in the uterus and pelvis.

KTS disproportionately affects single limbs, particularly lower extremities, though vascular malformations can be present anywhere in the body, including the pelvis, along the spine, within the spinal canal, and along the airway [3]. Larson and colleagues noted in their single-center study of 116 patients that among patients with KTS who underwent magnetic resonance imaging (MRI) neuroimaging, spinal neurovascular anomalies were observed in 16.4% of the patients, with four patients having multiple anomalies, including paraspinal/epidural venous malformations and epidural arteriovenous fistulas [5]. Little is known about the prevalence of vascular malformations in the airway. While proper evaluation and imaging are essential to developing an anesthetic plan in the parturient affected by KTS, this may be challenging in the unpredictable peripartum setting. We report a unique case of a parturient with KTS, diagnosed late in the second trimester, with a prominent facial port-wine stain, who underwent a successful emergent cesarean section under spinal anesthesia despite the absence of available imaging. This case highlights the safe use of neuraxial anesthesia to mitigate airway risks when a late diagnosis and urgent fetal distress preclude additional workup.

## Case presentation

A 35-year-old woman, G3P0020 at 32 weeks and four days, presented to the labor and delivery unit after referral from an outside clinic for the evaluation of pre-eclampsia with severe features. The patient’s past medical history was notable for a 10-year history of chronic hypertension that had previously never been addressed prior to this pregnancy. While receiving prenatal care at an outside clinic, she was referred to our maternal-fetal medicine (MFM) service at 20 weeks in the setting of chronic hypertension; advanced maternal age; a congenital port-wine stain along her entire face, including the lips, cheek, nose, eyelid, and forehead; and early-onset glaucoma at the age of 10, warranting genetics consultation.

Additional pertinent physical findings included varicose veins along her legs and soft tissue asymmetry, particularly mild hypertrophy of the right hip, gluteal, and leg tissues. Following evaluation by MFM and genetics at 23 weeks, she was diagnosed with Klippel-Trenaunay syndrome. Recommended management included the initiation of 40 mg of subcutaneous enoxaparin daily through six weeks post-delivery for thrombosis prophylaxis, imaging studies (maternal echocardiogram, magnetic resonance imaging of the brain, and renal ultrasound), maternal karyotype, and anesthesia consult prior to delivery. However, the patient was subsequently lost to follow-up with MFM at this time and did not undergo the recommended further work-up, including an anesthesiologist consultation.

Upon presentation to the labor and delivery unit at 32 weeks and four days, she continued to have persistently elevated blood pressures. The patient was admitted and started on intravenous magnesium, antenatal steroids, and labetalol. Within six hours of arrival, an emergency cesarean section was called by her obstetrics team for non-reassuring fetal heart rate tracing and concern for significant fetal distress.

On physical examination, no superficial vascular malformations were detectable along her lumbar spine. In contrast, a large, asymmetrical vascular malformation was noted around her lip, which extended along the upper and lower face, despite a reassuring airway examination with a Mallampati score of 2 and normal thyromental distance. No vascular malformations were visualized in the oral pharynx. Laboratory studies were within normal limits (Table 1).

**Table 1:** Baseline Laboratory Values

Laboratory	Values	Reference Range and Units
Hemoglobin	12.9	12.0-16.0 g/dL
Platelet Count	174	130-400 K/mm^3^
Creatinine	0.7	0.51-1.17 mg/dL
Prothrombin Time	10.2	9-11.5 Seconds
Partial Thromboplastin Time	28	23-32 Seconds
International Normalized Ratio	1.0	0.9-1.1
Fibrinogen	415	175-425 mg/dL

A multidisciplinary discussion was held with the patient and the obstetrics team concerning the optimal anesthetic plan. Considerations at this time included the possibility of a difficult airway due to an unidentified vascular malformation with cutaneous involvement, concern for the damage of vascular structures with laryngoscopy, and the potential for an undiagnosed, subglottic vascular malformation that could rupture during intubation. The patient’s last dose of enoxaparin was administered more than 12 hours prior, permitting the safe administration of neuraxial anesthesia. Eventually, the decision was made to proceed with cesarean section under spinal anesthesia, which would avoid the instrumentation of the airway and allow the patient to participate in the birth experience.

Spinal anesthesia was administered on a single attempt with a 25-gauge spinal needle to reduce the risk of damaging potential vascular structures or creating a significant neuraxial hematoma from a low-flow, venous malformation. No complications were noted, including no blood intermixed with cerebral spinal fluid or abnormal paresthesias. The patient subsequently underwent successful delivery under neuraxial anesthesia with no concerning back pain and motor or sensory deficits observed following delivery or prior to discharge. The patient was discharged from the hospital one week later.

## Discussion

Although there have been previous case reports of successful deliveries of parturient patients with KTS under neuraxial anesthesia, these cases remain rare, and reports are relatively limited [6-8]. In all three cases, MRIs were completed that were negative for spinal vascular malformations, though one MRI was completed six years earlier. None of the patients had visible vascular malformations overlying their lumbar spine. In contrast, Sivaprakasam and Dolak discuss a case of a parturient with fetal breech presentation, evolving pre-eclampsia, with the physical examination notable for prominent vascular malformations extending to the 11th thoracic dermatome. No spine imaging was available. After an unsuccessful external version and failure to progress, the decision was made to proceed with cesarean delivery under general anesthesia, which was uneventful [9].

Our case report is unique in three aspects. Firstly, KTS is largely diagnosed in childhood, with the median age of diagnosis at 11.9 years, and 91% of individuals have physical features of KTS at birth [10]. This patient’s diagnosis during pregnancy at 23 weeks of gestation and her eventual delivery at 32 weeks posed a significant challenge for the completion of imaging and the coordination of multiple specialties. Secondly, the presence of facial and cutaneous vascular anomalies is very uncommon but poses a theoretical risk of bleeding under general anesthesia with airway instrumentation. Lastly, in contrast to previous reports where patients underwent full pre-delivery evaluations, our patient’s emergent presentation required immediate action in the absence of a prior diagnostic workup.

Presently, there are no studies that have systematically investigated the correlation between superficial capillary malformations and underlying deep venous malformations. In a cohort of patients with KTS undergoing spinal MRI, 16.4% had spinal neurovascular anomalies, though the presence of overlying capillary malformations was not reported [5]. It is estimated that 98% of patients with KTS have capillary malformations compared to 72% with known venous malformations, suggesting that superficial malformations do not always correspond with deeper ones [10]. Nonetheless, a theoretical risk remains. KTS is part of a spectrum of disorders caused by mutations in the *PIK3CA* gene, which promote both tissue overgrowth and vascular malformations. In KTS, the mutation occurs in a mosaic pattern, leading to segmental involvement. Therefore, cutaneous findings can signal an increased risk of deeper vascular malformations within the same anatomical region [11]. Ultimately, these anomalies are complex and cannot be fully characterized in the absence of imaging.

KTS remains rare, and thus, no large consensus guidelines exist. Based on the current literature of case reports and studies, the management of KTS in pregnancy should include a multidisciplinary approach, detailed medical history and physical examination, imaging to characterize vascular malformations, coagulation laboratory tests, and thromboprophylaxis. We propose that all patients with KTS in the obstetric population should also be referred for anesthesia consultation, as well as have spinal imaging (particularly spinal magnetic resonance imaging and angiogram as it pertains to neuraxial anesthesia) ordered as part of routine care.

## Conclusions

In urgent situations where prior spine imaging is not possible, neuraxial anesthesia may still be an appropriate anesthetic option when weighing risks and benefits. In our case, general anesthesia was deemed a greater risk to the patient than neuraxial anesthesia in the absence of visible capillary malformations over the lumbar spine and the presence of these anomalies throughout the face. Ultimately, extensive multidisciplinary discussions with patients and providers are essential in determining the best anesthetic plan for each patient.
